# Ammonium Recovery and Biogas Upgrading in a Tubular Micro-Pilot Microbial Electrolysis Cell (MEC)

**DOI:** 10.3390/molecules25122723

**Published:** 2020-06-12

**Authors:** Lorenzo Cristiani, Marco Zeppilli, Cristina Porcu, Mauro Majone

**Affiliations:** Department of Chemistry, University of Rome Sapienza, Piazzale Aldo Moro 5, 00185 Rome, Italy; marco.zeppilli@uniroma1.it (M.Z.); Porcu.1420532@studenti.uniroma1.it (C.P.); mauro.majone@uniroma1.it (M.M.)

**Keywords:** biogas upgrading, nitrogen recovery, microbial electrolysis cell, bioelectromethanogenesis, digestate

## Abstract

Here, a 12-liter tubular microbial electrolysis cell (MEC) was developed as a post treatment unit for simultaneous biogas upgrading and ammonium recovery from the liquid effluent of an anaerobic digestion process. The MEC configuration adopted a cation exchange membrane to separate the inner anodic chamber and the external cathodic chamber, which were filled with graphite granules. The cathodic chamber performed the CO_2_ removal through the bioelectromethanogenesis reaction and alkalinity generation while the anodic oxidation of a synthetic fermentate partially sustained the energy demand of the process. Three different nitrogen load rates (73, 365, and 2229 mg N/Ld) were applied to the inner anodic chamber to test the performances of the whole process in terms of COD (Chemical Oxygen Demand) removal, CO_2_ removal, and nitrogen recovery. By maintaining the organic load rate at 2.55 g COD/Ld and the anodic chamber polarization at +0.2 V vs. SHE (Standard Hydrogen Electrode), the increase of the nitrogen load rate promoted the ammonium migration and recovery, i.e., the percentage of current counterbalanced by the ammonium migration increased from 1% to 100% by increasing the nitrogen load rate by 30-fold. The CO_2_ removal slightly increased during the three periods, and permitted the removal of 65% of the influent CO_2_, which corresponded to an average removal of 2.2 g CO_2_/Ld. During the operation with the higher nitrogen load rate, the MEC energy consumption, which was simultaneously used for the different operations, was lower than the selected benchmark technologies, i.e., 0.47 kW/N·m^3^ for CO_2_ removal and 0.88 kW·h/kg COD for COD oxidation were consumed by the MEC while the ammonium nitrogen recovery consumed 2.3 kW·h/kg N.

## 1. Introduction

Biogas, the main product of the anaerobic digestion (AD) process, is a gas mixture mainly composed of carbon dioxide and methane [1,2]. To obtain biomethane with a high percentage of methane (>95%), an upgrading operation to increase the CH_4_ content through CO_2_ removal and a purification step aimed at impurity removal (NH_3_, H_2_S) are necessary to increase the gas mixture calorific power [3,4,5]. Due to the investment and operations costs required for the purification and upgrading steps, biogas is commonly utilized for the cogeneration of electricity and heat through the CHP (Combined Heat Power) unit; however, due to the recent emission reduction goals stated by the European Union for 2050 [6,7], an incentive plan for biogas conversion into methane has recently been activated in different European countries [8]. Biomethane can be considered as a renewable carbon neutral fuel with high added value, which can be used in automotive engines or injected into the natural gas grid [9,10]. In order to couple CO_2_ emission mitigation and renewable energy storage [11], several approaches for biogas upgrading have been proposed in the literature [12]; basically, the biological approach for biogas upgrading consists in the supply of renewable hydrogen to methanogens, which are able to convert CO_2_ into CH_4_ [13,14]. 

Along the different hydrogen supply techniques, which includes in situ [15] and ex situ approaches [16], the use of bioelectrochemical systems to supply the reducing power resulted in a more sustainable approach due to the utilization of mild reaction condition as well as the use of a robust and low-cost catalytic material widely present in the AD processes [17]. The bioelectrochemical system exploits the ability of the electroactive microorganisms to exchange electrons with solid electrodes by the extracellular electron transfer mechanism (EET) [18]. The interphase constituted by an electroactive biofilm on an electrode can be named a bioelectrode [19]; in more detail, when the electroactive biofilm uses the electrode as an electron acceptor, the electrochemical interphase acts as a bioanode [20]; on the contrary, if the electroactive biofilm uses the electrode as an electron donor, the interphase is defined as a biocathode [21]. The electron exchange between the electroactive biofilm and the electrodic material can be directly performed by specialized membrane proteins or by the utilization of mediators, which have the function of electrons shuttles between the biofilm and the electrode surface [22,23]. Biocathode utilization has been investigated for several environmental applications, which includes biofuel production [24,25], CO_2_ fixation into VFA (Volatile Fatty Acid) [26,27], and groundwater bioremediation [28,29]. The bioelectrochemical reduction of CO_2_ into CH_4_, named the bioelectromethanogenesis reaction, is obtained by using an electrodic material for the reducing power supply to mixed methanogenic consortium, which adopts the electrodic material as an electron donor. Two limit mechanisms regulate the electrode–microorganisms interaction, i.e., a direct electron uptake [30] and a hydrogen-mediated [31] mechanism have been identified; however, specifically for the bioelectromethanogenesis reaction, several intermediate steps for the electrode–microorganisms interaction have recently been reported in the literature [32]. The utilization of the bioelectromethanogenesis reaction requires the utilization of a microbial electrolysis cell (MEC), in which, by the application of an external potential, partial energy support is supplied by the anodic bioelectrochemical oxidation of organic waste streams [33,34]. Several authors proposed the utilization of MECs for biogas upgrading into biomethane with different configurations, including the direct treatment of biogas [35,36] or separate conversion of the residual CO_2_ from the upgrading step in the biocathode [37]. Moreover, in an MEC biocathode, the main CO_2_ removal mechanism along with the bioelectromethanogenesis reaction is represented by the CO_2_ sorption as HCO_3_^−^ promoted by alkalinity generation, which directly depends on the transport of ionic species different from protons and hydroxyls for the maintenance of electroneutrality [38], i.e., the alkalinity generation in an MEC biocathode permits the removal of up to 9 moles of CO_2_ for each mole of CH_4_ produced [39]. 

When using a cation exchange membrane as a separator in an MEC, which receives an anolyte with a physiological pH, the electroneutrality maintenance is ensured by several cations different from protons, such as the ammonium ion, which is considerably present in the anaerobic digestion liquid effluents [40]. In an MEC, it is possible to exploit the migration of the ammonium ion caused by the electroneutrality maintenance as a mechanism to recover ammonium nitrogen [41]. Ammonium nitrogen is usually present at high concentrations in manure and digestate due to the proteins’ hydrolysis [42]. The integration of the AD process and an MEC has been tested by using real effluents as anodic substrates of a methane-producing MEC [43], which allowed ammonium recovery and CO_2_ removal in the biocathode. Moreover, a new three-chamber configuration MEC with a two-sided cathode configuration was successfully tested [44]. Even if the bioelectromethanogenesis reaction and ammonium recovery are well known at the laboratory scale, with several configurations, few studies have reported scale-up attempts of the MEC process. In the present study, a 12-L micro-pilot MEC [45] with a tubular geometry was designed for the integration of the process with a two-stage anaerobic digestion process in which the biogas upgrading through the bioelectromethanogenesis reaction is coupled with the oxidation of COD and nitrogen recovery in the anodic chamber. A synthetic feeding solution containing a mixture of VFA was used as the substrate of the anodic bioelectrochemical oxidation, while three different ammonium nitrogen load rates (73, 365, and 2229 mg·N/Ld) were tested to assess the process performances and possible poisoning effects of the high ammonium concentration. The three nitrogen load rates were chosen following previous experiments performed in a bench-scale filter press MEC [40,43]. The bioelectrochemical process was evaluated by the analysis of the COD removal, CH_4_ production and CO_2_ removal, and ammonium nitrogen migration and recovery.

## 2. Materials and Methods 

### 2.1. Micro-Pilot MEC Set Up

**Figure 1:**
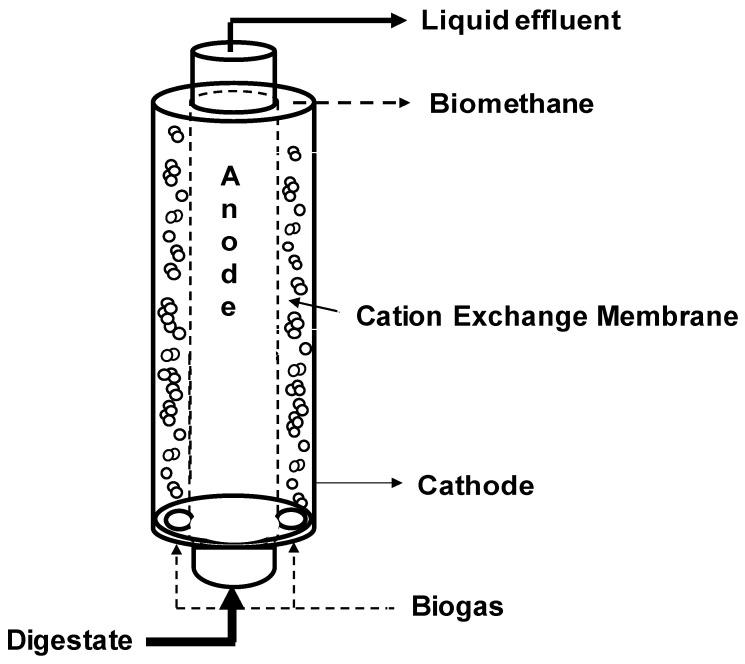
Scheme of the tubular microbial electrolysis cell.

The micro-pilot MEC was built starting from a 12-L plexiglass cylindric reactor, and dividing it with a 2355 cm^2^ cation exchange membrane (CEM) Fumasep FKS-PET reinforced 75-µm Fumatech (Bietigheim-Bissingen, Germany) in two concentric chambers (Figure 1). Both chambers were filled with granular graphite with a porosity of 0.57 and a surface area of 1290 m^2^/m^3^. The outer chamber (cathodic chamber, total volume of 8.86 L, working volume of 5.06 L) was equipped with an external glass chamber for liquid and gas sample collection placed above the tubular MEC and connected with a Tygon R3603 pipe (Saint-Gobain, Courbevoie, France), while the inner chamber (anodic chamber, volume of 3.14 L) was equipped with an external glass sampling cell for liquid sample collection, placed also above the tubular MEC and connected with a Tygon R3603 tube (Saint-Gobain). The anodic chamber was inoculated with 2 L of prewashed activated sludge (8.3 g VSS/L) coming from a full-scale wastewater treatment plant in Treviso (Italy). During the start-up period and during the experimentation, the anodic potential was controlled at +0.2 V vs. SHE to select the electroactive microorganism capable of using the electrode as the final electron acceptor. Meanwhile, the cathodic chamber was inoculated with 3 L of a prewashed anaerobic sludge (7.5 g VSS/L) from a pilot-scale anaerobic digester. The anodic chamber was continuously recirculated with a peristaltic pump, and periodically fed with a volatile fatty acids (VFAs) mixture with an average pH value of 7.1 ± 0.1 composed by: Sodium acetate (0,47 g/L), propionic acid (0.17 g/L), and butyric acid (0.14 g/L) added to the mineral medium (NH_4_Cl [0.125 g/L], MgCl_2_ 6H_2_O [0.1 g/L], K_2_HPO_4_ [4 g/L], CaCl_2_ 2H_2_O [0.05 g/L], 10 mL/L of a trace metal solution, and 1 mL/L of a vitamin solution). After the start-up period, the anodic chamber was continuously fed with a peristaltic pump with a loading rate of circa 7 L/day. The cathodic chamber was continuously fed with a gaseous mix composed of 30% (*v*/*v*) CO_2_ and 70% N_2_ to simulate a CO_2_ concentration comparable to a biogas CO_2_ content with a flow rate of 45 L/day. The catholyte solution was never replaced and was composed by NH_4_Cl (0.125 g/L), MgCl_2_ 6H_2_O (0.1 g/L), K_2_HPO_4_ (4 g/L), CaCl_2_ 2H_2_O (0.05 g/L), 10 mL/L of a trace metal solution, and 1 mL/L of a vitamin solution. The cathodic chamber was continuously recirculated with a peristaltic pump while the anodic liquid phase electroosmotic diffusion through the CEM membrane required a daily spill from the cathodic chamber. A three-electrode configuration was adopted by using an AMEL model 549 potentiostat and an Ag/AgCl electrode (+0.2 V vs. SHE) was used as a reference electrode. During all the experimental period, the anodic chamber resulted in the working electrode while the cathodic the counter electrode. A digital multimeter (Aim-TTI 1604) was connected to the circuit to measure the potential difference between the two electrodes (ΔV). The operational temperature was the laboratory temperature, controlled around 25 °C.

### 2.2. Analytical Methods

The chemical oxygen demand (COD) inside the liquid samples was calculated by converting the measured concentration of the VFAs (acetic acid, propionic acid, and butyric acid). The VFAs determination was performed by injecting 1 µL of prefiltered sample (the anolyte, catholyte, and anodic influent were sampled each with a 10-mL plastic syringe, 0.2 µm, from FARMAC-ZABBAN, Bologna, Italy) into a Dani Master GC (stainless-steel column packed with a molecular sieve; He as the carrier gas 18 mL/min; oven temperature 175 °C; flame ionization detector (FID) temperature 200 °C), from DANI Analitica, Milan, Italy. The methane content inside the gas phase was analyzed by sampling 10 μL of the headspace by a gas-tight Hamilton syringe and injecting it into a Dani Master gas-chromatograph stainless-steel column packed with a molecular sieve; He as the carrier gas 18 mL/min; oven temperature 50 °C; flame ionization detector (FID) temperature 200 °C. The H_2_ and the CO_2_ determination were performed by injecting 50 µL of gaseous sample into a Dani Master GC (stainless-steel column packed with a molecular sieve; He as the carrier gas 18 mL/min; oven temperature 70 °C; thermal-conductivity detector (TCD) temperature 200 °C). The inorganic carbon was measured by TOC (Total Organic Carbon Analyzer)-V CSN (Shimadzu, Kyoto, Japan) on filtered samples (0.2 μm). The VSS were measured using GF/C filter (47 mm diameter, 1 µm porosity) following the standard (1992) procedure [46]. The Nessler method was used to determine spectrophotometrically (420 nm) the concentration of ammonium ion [46].

### 2.3. Calculations

The COD removal was calculated as the difference between the amount of COD entering the system and the outgoing quantity of COD (mg/d), according to Equation (1):

(1) $${\mathrm{COD}}_{\mathrm{removed}\;\left(\frac{\mathrm{mg}}{d}\right)}=F_{\mathrm{in}}\times {\mathrm{COD}}_{\mathrm{in}}-F_{\mathrm{out}}\times {\mathrm{COD}}_{\mathrm{out}},$$

where COD_in_ (mg/L) and COD_out_ (mg/L) are the COD concentration in the inlet and outlet of the anodic chamber, respectively. Moreover, F_in_(L/d) and F_out_(L/d) are the influent and effluent flow rates in the anodic chamber. Furthermore, the COD removal efficiency was calculated as:

(2) $${\mathrm{COD}}_{\mathrm{removal}\;\mathrm{efficiency}\;\left(\%\right)}=\frac{F_{\mathrm{in}}\times {\mathrm{COD}}_{\mathrm{in}}-F_{\mathrm{out}}\times {\mathrm{COD}}_{\mathrm{out}}}{F_{\mathrm{in}}\times {\mathrm{COD}}_{\mathrm{in}}}\times 100.$$

The COD oxidation reaction equation can be written as follows:

(3) $$C_{x}H_{y}O_{z}N+\left(2x-z\right)H_{2}O\;\underset{}{\to }{\mathrm{xCO}}_{2}+\left[y+\left(2x-z\right)\right]\;\left[e^{-}+H^{+}\;\right]+{\mathrm{NH}}_{3}.$$

Keeping in mind the water oxidation reaction, the quantity of COD diverted into the electric current is also expressed as equivalents of electrons:

(4) $$2H_{2}O\;\underset{}{\to }O_{2}+4e^{-}+4H^{+}.$$

The coulombic efficiency (CE%) represents the fraction of oxidized COD converted into electric current. It was calculated by converting the flowing current and the COD into equivalents of electrons:

(5) $$\mathrm{CE}\;\left(\%\right)=\frac{{\mathrm{meq}}_{i}}{{\mathrm{meq}}_{\mathrm{COD}}}\times 100.$$

The flowing current was converted into equivalents by integrating the current over time and dividing it by the Faraday’s constant (F = 96485 C/mol_e_−).

The methane production rate (rCH_4_, mmol/d) was expressed in equivalents considering the conversion factor of 8 eq/mol, which was calculated taking into account the following equation:

(6) $${\mathrm{CO}}_{2}+8e^{-}+8H^{+}\;\underset{}{\to }{\mathrm{CH}}_{4}+2H_{2}O,$$

(7) $${\mathrm{rCH}}_{4\left(\mathrm{mmol}\right)}\times 8={\mathrm{rCH}}_{4\left(\mathrm{meq}\right)}.$$

The cathodic capture efficiency (or cathodic coulombic efficiency, CCE) represents the fraction of electric current converted into methane. It was expressed as a ratio between the cumulative equivalents of the produced methane and the equivalents of the current flowing in the circuit in the same amount of time:

(8) $$\mathrm{CCE}\left(\%\right)=\frac{{\mathrm{meq}}_{CH4}}{{\mathrm{meq}}_{i}}\times 100.$$

The energetic efficiency (ηE) of the process was measured. This efficiency expresses the ratio between the recovered energy from the combustion of methane and only the spent energy for the polarization of the MEC (excluding the other operational costs):

(9) $$\eta E\left(\%\right)=\frac{n_{CH4}\times \Delta G_{CH4}}{\Delta V\times i_{A}}\times 100.$$

### 2.4. Inorganic Carbon Mass Balance 

The daily removal of carbon dioxide (ΔCO_2_, mmol/day) was calculated using the following equation:

(10) $$\Delta {\mathrm{CO}}_{2}=Q_{\mathrm{in}}\times {\mathrm{CO}}_{2\;\mathrm{in}}-Q_{\mathrm{out}}\times {\mathrm{CO}}_{2\;\mathrm{out}},$$

where Q_in_ and Q_out_ are the gaseous influent and effluent flowing rate (L/day), respectively. Moreover, CO_2 in_ and CO_2 out_ are the concentrations of carbon dioxide inside the cathodic gaseous inlet and outlet, respectively. Many species of inorganic carbon (i.e., HCO_3_^−^ and CO_2_) and the carbon dioxide reduction to methane were taken into consideration for the mass balance equation:

(11) $$Q_{\mathrm{in}}\times {\mathrm{CO}}_{2\;\mathrm{in}}=Q_{\mathrm{out}}\times \left({\mathrm{CO}}_{2\;\mathrm{out}}\right)+r_{CH4\left(\mathrm{mmol}\right)}+F_{\mathrm{spill}}\times {\mathrm{HCO}}_{3\mathrm{spill}}^{-},$$

where Q_in_ and Q_out_ are the gaseous influent and effluent flowing rate (L/day), respectively. Moreover, CO_2 in_ and CO_2 out_ are the concentrations of carbon dioxide inside the cathodic gaseous inlet and outlet; F_spill_ was the amount of daily spilled liquid from the cathodic chamber; HCO_3_^−^_spill_ represents the bicarbonate concentration inside the daily spill; and r_CH4_ is the methane production rate.

### 2.5. Nitrogen Mass Balance

The daily nitrogen removal (ΔN; mg/day) was evaluated by the following equation:

(12) $$\Delta N_{}=F_{\mathrm{in}}\times N_{\;\mathrm{in}}-F_{\mathrm{out}}\times N_{\;\mathrm{out}},$$

where F_in_ and F_out_ (L/d) are the influent and effluent liquid flow rates, respectively. Moreover, N_in_ and N_out_ (mg/L) represent the nitrogen concentration inside the inlet and outlet of the anodic chamber.

Since the nitrogen was in the form of ammonium, it could migrate through the CEM and it was detected inside the cathodic chamber, where it was recovered inside the daily spill. A small portion of ammonium is used by microorganisms for reproduction. This was taken into consideration for the mass balance equation according to the generic biomass composition (C_5_H_7_O_2_N):

(13) $$\;F_{\mathrm{in}}*N_{\mathrm{in}}=F_{\mathrm{spill}}\times \left(N_{\mathrm{Cat}}\right)+F_{\mathrm{out}}\times \left(N_{\mathrm{out}}^{}+{\mathrm{VSS}}_{\mathrm{out}}\times 0.12\right),$$

where F_in_ and F_out_ (L/d) are the influent and effluent liquid flow rates, respectively. Moreover, N_in_ and N_out_ (mg/L) represent the nitrogen concentration inside the inlet and outlet of the anodic chamber. N_cat_ represents the nitrogen concentration (mg/L) inside the cathodic chamber and F_spill_ is the daily spill (L/day) from the cathodic chamber; VSS_out_ is the measured concentration (mg/L) of the volatile suspended solid inside the anodic effluent, and 0.12 is the conversion factor used for determining the ammonium nitrogen used for the biomass reproduction (mg·N/mg VSS). Moreover, the nitrogen contribution to the total charge transport inside the MEC was calculated by using the following equation:

(14) $$i_{\mathrm{ionic}}=\frac{\left[{\mathrm{NH}}_{4}^{+}\right]\times F_{\mathrm{spill}}\times Z\times F}{86400s},$$

where F_spill_ represents the daily spill (L/day) from the cathodic chamber, and [NH_4_^+^] is the ammonium concentration (mol/L) inside the cathodic chamber. Z is the amount of charge transported by the cation, F is the Faraday’s constant (96,485 C/mol_e_−), and 86,400 is the seconds in one day.

## 3. Results and Discussion

### 3.1. Electrodic Reaction’s Performances

**Figure 2:**
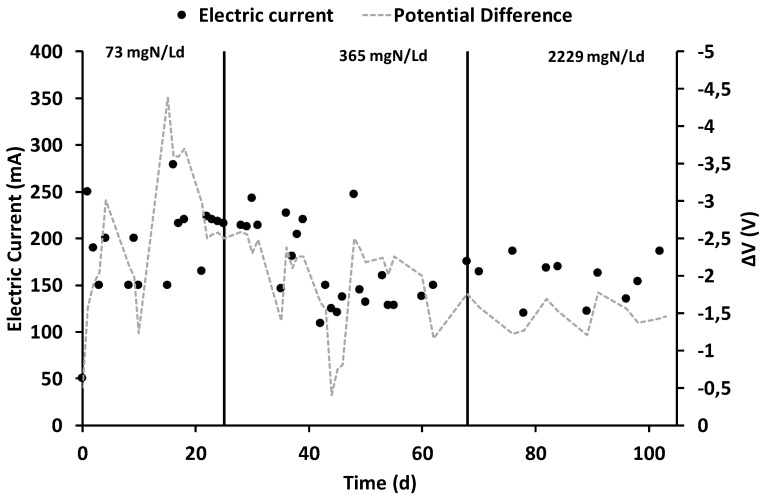
Electric current and potential difference’s trends during the three different operating periods.

**Figure 3:**
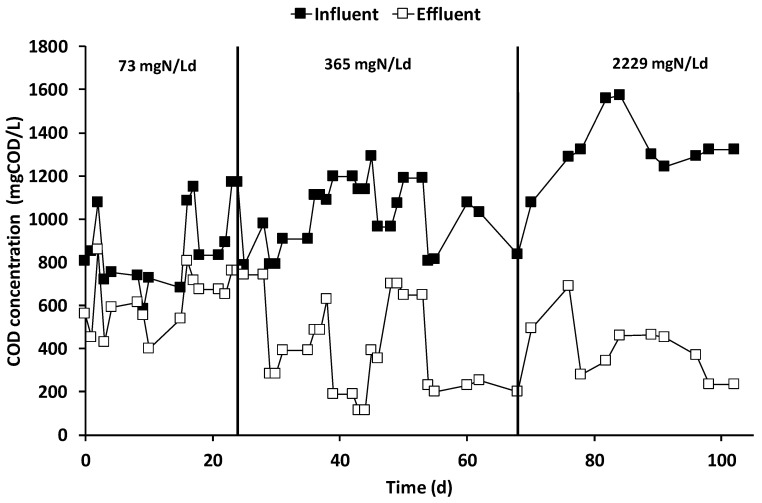
Influent and effluent COD concentration in the anodic chamber of the MEC during the three operational periods.

**Figure 4:**
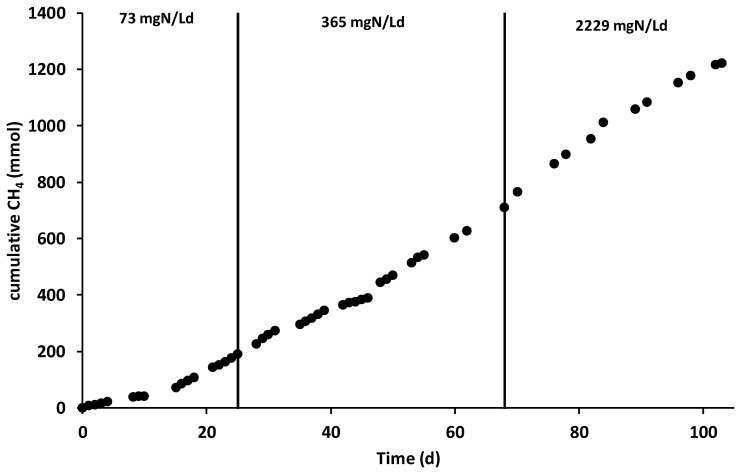
Cumulative methane production during the three different operating periods.

After the inoculation and the consequent start-up period (which lasted 15 days), characterized by the polarization of the anode chamber at +0.20 V vs. SHE, the increase in current generation indicated the electroactive biofilm’s formation, which oxidized the organic substrates using the graphite granules as final electron acceptors. During all the operating periods, the anodic chamber was continuously fed with the VFAs synthetic mixture with an average flow rate of 6.9 ± 0.2 L/day, corresponding to a hydraulic retention time (HRT) of 0.52 days. During the first operating period, the theoretical ammonium concentration was 32 mg·N/L corresponding to a nitrogen load rate of 73 mg·N/Ld. As reported in Figure 2, the average electric current was 190 ± 14 mA, which was generated by an average COD removal of 1.8 ± 0.3 g COD/day (Figure 3), corresponding to a COD removal efficiency of 29 ± 11%. The fraction of COD transformed into electric current, named the coulombic efficiency (CE), was on average 77 ± 18%. The main product detected in the cathodic chamber, as reported in Figure 4, was methane, with an average production rate of 9 ± 1 mmol/day. The fraction of electric current converted into methane, defined as the cathodic capture efficiency (CCE), was on average 42 ± 8%, which indicated the, low activity of the methanogens probably due to their long acclimatization time. During the first run, as reported in Figure 2, the average cell voltage measured between the anode and cathode was −2.66 ± 0.25 V.

After 25 days, the nitrogen concentration inside the feeding solution was increased five times, giving a theoretical nitrogen loading rate of 365 mg·N/Ld. During this second run, as reported in Figure 2, an average electric current of 166 ± 10 mA was obtained with a consumption of 4.0 ± 0.3 g COD/day, giving a CE average value of 30 ± 4%. As showed in Figure 3, the COD removal efficiency increased to 65 ± 17% probably due to non-electroactive microorganisms’ activity, which were previously inhibited by the low concentration of nitrogen in the anodic chamber. Inside the cathodic chamber, as reported in Figure 4, an increase in the methane production rate to 18 ± 1 mmol/day was obtained, giving an average value of CCE 98 ± 11%, indicating an almost complete utilization of the current for CO_2_ reduction into CH_4_. The cell voltage applied between the anode and cathode was on average −2.00 ± 0.13 V during the second operating condition of the MEC.

After 40 days, the nitrogen concentration inside the feeding solution was raised to a theoretical concentration of 1000 mg·N/L, corresponding to a nitrogen load rate of 2229 mg·N/Ld. During the run with the higher ammonium concentration, reported in Figure 2, an average value of electric current of 157 ± 7 mA was obtained, while the COD removal shown in Figure 3 increased to 6.3 ± 0.6 g COD/day. The increase in COD removal, without a corresponding increase in terms of the electrical current, affected the CE of the process, which was only 18 ± 2%. The increase of the COD removal efficiency up to 70% probably indicated an underestimation of the biomass growth in the anodic chamber or the presence of non-electroactive COD removal pathways like COD sorption or entrapment in the biofilm matrix [47]. 

As reported in Figure 4, the methane production rate was almost stable, with an average value of 14 ± 2 mmol/day, which resulted in an average CCE value of 81 ± 14%. No inhibition effect of the high ammonium concentration was detected during the last run of the MEC. The cell voltage (Figure 2) obtained in the last operating period was −1.48 ± 0.08 V. Table 1 summarizes all the main parameters describing the performances of the bioelectrochemical reactions in the three different operating periods.

### 3.2. NH_4_^+^ Removal and Nitrogen Mass Balance

**Figure 5:**
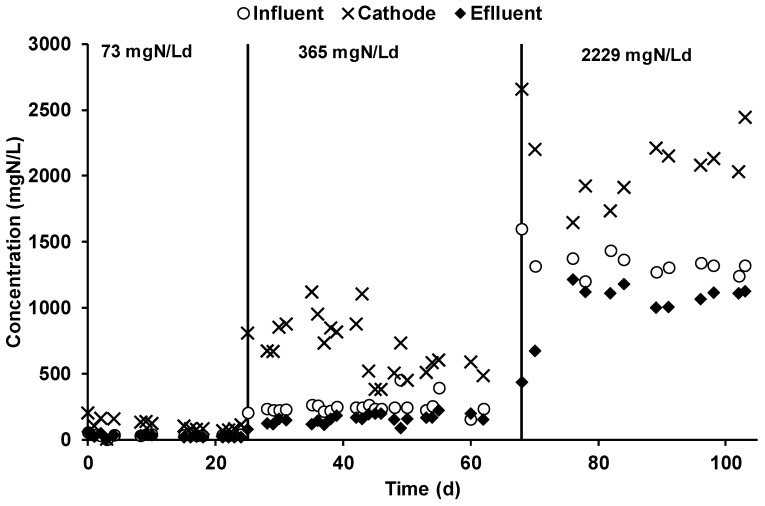
Ammonium concentration inside the chambers and the inlet of the anodic chamber.

The ammonium nitrogen concentration was monitored in all of the reactor streams. As reported in Figure 5, during the first operating period, the average influent ammonium concentration was 37 ± 2 mg·N/L, while the average effluent ammonium concentration was 25 ± 2 mg·N/L; on average, 89 ± 31 mg·N/day were removed, giving a corresponding nitrogen removal efficiency of 33 ± 13%. 

The ammonium was mainly removed through its migration through the CEM membrane, i.e., an ammonium concentration of 101 ± 9 mg·N/L in the cathodic chamber underlined the migration of ammonium ions, which result in a 4 times higher concentration with respect to the anodic concentration. The steady-state achievement was underlined by the stable concentration of ammonium, which was caused by the daily catholyte spill performed to counterbalance the electroosmotic diffusion phenomenon. During the second operating period, the influent and effluent ammonium concentration in the anodic chamber was 241 ± 14 and 148 ± 9 mg·N/L, respectively. A nitrogen removal efficiency of 45 ± 12% was obtained by the daily removal of 713 ± 150 mg·N/day. The concentration of the ammonium in the cathodic chamber (Figure 5) was 674 ± 48 mg·N/L, a 4.5 times higher value with respect to the anodic ammonium concentration. By applying the higher nitrogen load rate, which corresponded to an average anodic influent ammonium concentration of 1341 ± 28 mg·N/L, the daily nitrogen removal was on average 3246 ± 558 mg·N/d due to an anodic effluent concentration of 1013 ± 66 mg·N/L. The resulting nitrogen removal efficiency was quite like the previous operating periods, with an average value of 36 ± 7%. The concentration of the ammonium ion in the catholyte during the last operating condition was 2094 ± 78 mg·N/L. Table 2 summarizes all the average ammonium concentrations observed in the different MEC streams during the three different operating conditions. An average VSS cathodic concentration of 62 ± 2 mg VSS/L was also determined, which resulted in a negligible amount of fixed nitrogen. The ammonia content in the outcoming gas was occasionally monitored by an acid trap, which was placed at the end of the gas pipeline. No ammonia was ever detected in the outcoming gas, during all of the experimental period. 

The nitrogen mass balance, which is summarized in Table 2, reports the two main removal mechanisms detected for the removal of the ammonium ion from the anodic feeding solution. The biomass formation, evaluated by the determination of the volatile suspended solids (VSSs) in the anodic effluent, and the migration and the consequent daily spill of the cathodic liquid phase. This last procedure was the main ammonium removal mechanism involved in the process. During the three different operating periods, the daily spill of catholyte permitted the recovery of 31 ± 3, 281 ± 20, and 2445 ± 9 mg·N/day. A significant increase of the cathodic spill flow rate was observed during the three operational periods, i.e., the cathodic spill flow rates were 0.31 ± 0.02, 0.42 ± 0.08, and 1.17 ± 0.20 L/day. The change in the cathodic spill flow rate was reasonably explained by the analysis of the ammonium contribution to the electroneutrality maintenance, i.e., while in the first two conditions, 1% and 22% of the current was counterbalanced by the ammonium ion; in the third operating condition, almost all the current was transported by the ammonium ion. Interestingly, the increase of the ionic current transported by the ammonium ion was not linear to the influent concentration, i.e., the 5-fold concentration increase in the second period corresponded to an increase of 22-fold while, by increasing the influent concentration 30 times, a percentage increase of 100%. 

The ammonium migrates from the anodic chamber to the cathodic one through the cation exchange membrane against the concentration gradient to maintain the electroneutrality of the chambers. The average concentration reached inside the cathodic chamber was 101 ± 9 mg·N/L. Moreover, the ammonium migration transported only1% of the ionic charge. Concerning the second stream, the nitrogen concentration was raised by five times, giving as a result an average concentration of 674 ± 48 mg·N/L inside the cathodic chamber. Furthermore, the total nitrogen removal was of 45% with a transported charge of 22 ± 2 mA (13%). During the third stream, considering a cathodic daily spill of 1.17 ± 0.20 L/day, the ammonium recovery was 2.5 ± 0.1 g·N/day, which was responsible for 195 ± 7 mA of transported charge, giving a 124% contribution to electroneutrality maintenance. 

### 3.3. CO_2_ Removal and Inorganic Carbon Mass Balance

**Figure 6:**
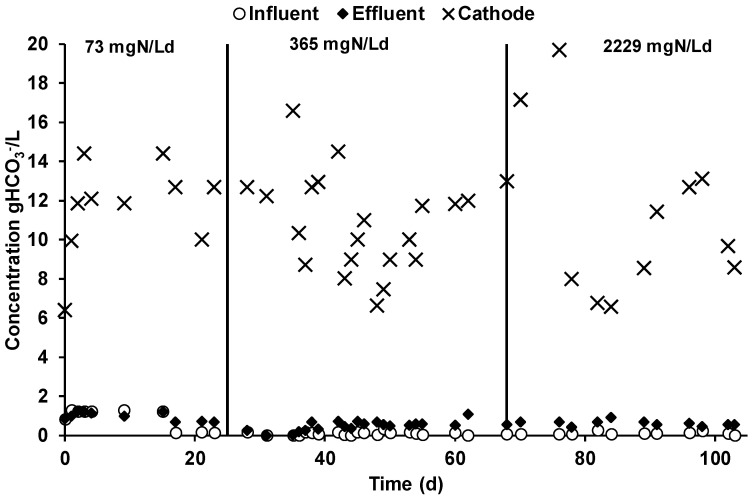
Bicarbonate concentration in the different MEC streams during the three different operating periods.

The daily CO_2_ removal obtained in the cathodic chamber was on average 443 ± 40, 453 ± 19, and 481 ± 38 mmol/day during the three different MEC operating periods. Those values were calculated by measuring the CO_2_ concentration difference between the influent and the effluent gas flow of the cathodic chamber. The bicarbonate concentration in the different reactor streams, reported in Figure 6, showed the effect of the alkalinity generation in the cathodic liquid phase, which promoted the sorption of bicarbonate at a higher concentration with respect to the anode chamber. As a result, the average cathodic pH value was 7.5 ± 1.

As reported in Table 3, during the three different operating periods, the cathodic bicarbonate concentration showed similar concentrations of 10.94 ± 1.20, 0.72 ± 0.56, and 11.39 ± 2.10 gHCO_3_^−^/L, while, due to the utilization of a CEM membrane which avoids bicarbonate diffusion, the influent and the effluent concentrations in the anodic chamber (Figure 6) were considerably lower with respect to the cathodic chamber. A slight increase in the bicarbonate concentration in the anodic effluent solution (Table 3) was detected during the second and third operational period due to the slight modification of the feeding solution preparation, which caused a decrease of its bicarbonate content; the bicarbonate increase was caused by the VFA oxidation. 

The analysis of the mechanisms involved in the CO_2_ removal during the three operating periods is reported in Table 3. The two mechanisms characterized during the operation resulted in methane production and bicarbonate removal within the daily cathodic liquid phase spill. In the first operating period, the methane production was 9 mmol/day, which corresponds only to 2% of the CO_2_ removal, while a bicarbonate spill of 55 ± 5 mmol/day resulted in 12% of the removed CO_2_. Similar results were obtained during the second and the third operating period, with higher methane production rates of 18 ± 1 and 14 ± 2 mmol/day, which contributes 4% and 3% of the CO_2_ removal, respectively. Moreover, during the latter two operating periods, the bicarbonate spill was 73 ± 4 and 218 ± 40 mmol/day, which corresponded to 16% and 45% of the removed CO_2_. As reported in the previous chapter, the substantially higher contribution of the bicarbonate spill during the third operating period resulted from the substantial increase in the cathodic spill flow rate, which increased the daily bicarbonate removal.

The high percentage of unjustified CO_2_ removed clearly indicates that other mechanisms contributed to the overall CO_2_ removal in the process. In this sense, a hypothesis can be elaborated for the CO_2_ removal justification. The CO_2_ removal in the cathodic chamber could be increased by the precipitation of low-soluble carbonates with alkaline earth metals, such as calcium and magnesium, which are present in the synthetic feeding solution and can be transported from the anode to the cathode by migration for the electroneutrality maintenance. By taking into account the current, which is not justified by the ammonium migration, previously reported in Table 2, a daily migration of 94 and 72 mmol/day of calcium and magnesium was evaluated for the first and the second operating period, respectively. Moreover, by assuming the complete precipitation of low-soluble carbonate salts, calcium and magnesium migration accounted for 21% and 16% of the CO_2_ removal in the first and second operating periods. During the third operating period, ammonium migration was the only cation responsible for the electroneutrality maintenance. Considering the calcium and magnesium migration and carbonate precipitation in the cathodic chamber, an overall recovery of 42%, 45%, and 48% of the removed CO_2_ was obtained in the three different operating periods. Moreover, even if the cathodic biomass concentration in the catholyte was only 62 ± 2 mg VSS/L, resulting in a negligible contribution to the cathodic CO_2_ removal, a possible underestimation of this mechanism can be present due to the high surface area of the cathodic chamber of the tubular MEC.

### 3.4. Energetic Consumption and Evaluation of the Process

The MEC energy consumption was calculated for the COD removal inside the anodic chamber, the CO_2_ removal in the cathodic chamber, and the energetic cost of the nitrogen recovery form the cathodic phase spill. The energetic consumption for each MEC operation was compared with a selected benchmark technology, i.e., activated sludge for COD removal and water scrubbing for biogas upgrading (expressed as CO_2_ removal). Concerning the energetic consumption for nitrogen recovery, the process of energy consumption was compared to the sum of the energetic cost for ammonium production (Haber Bosch process) and the nitrification/denitrification process in a wastewater treatment plant, which accounted for 8.5 and 12.5 kW·h/kg N, respectively. The energy consumption for the different operations, as reported in Table 4, decrease within the increase of the nitrogen load rate; this energy consumption reduction was caused by the increase of the reactor performances concerning COD and CO_2_ removal and nitrogen recovery.

The lowest energy consumption for COD and CO_2_ removal was obtained during the third operating period characterized by the higher nitrogen load rate, with an average value of 0.88 ± 0.08 kW·h/kg COD for COD removal and 0.47 ± 0.02 kW·h/Nm^3^ CO_2_ for the CO_2_ removal. The energetic cost of the nitrogen recovery was 2.3 ± 0.5 kW·h/kg N, a considerably lower value with respect to the production and removal of ammonium. It is also noteworthy to mention that the energy consumption in an MEC is adopted for the simultaneous operation of the COD and CO_2_ removal as well as for the nitrogen recovery, i.e., during the third operational period, with 0.47 kW·h/d 1 m^3^ of CO_2_ removed by the biocathode, while, at the same time, the MEC oxidized 0.53 kg/day of COD in the bioanode and 0.21 kg N/day was recovered as concentrated ammonium solution. Finally, at the biocathode, methane production resulted in additional energy recovery given by its energetic content, i.e., a theoretical energy efficiency of 59 ± 1% was also obtained during the third operating condition. 

### 3.5. Comparison of the Upscaled Process with the Previous Bench-Scale Reactor

The tubular MEC performances regarding the CO_2_ removal and ammonium recovery were compared with previous experiments [39,40,43] performed on a bench-scale MEC, which adopted a simple filter press configuration, in which the same bioelectrochemical reactions and similar operating conditions were adopted. The filter press MEC configuration presented a cathodic and anodic with an empty volume of 0.86 L while the tubular MEC presented in this study resulted in an empty volume of 3.14 and 8.86 L for the anodic and cathodic chamber, respectively. Regarding the cathodic CO_2_ removal, the tubular MEC was capable of removing on average 50 mmol CO_2_/Ld while in a previous study, 100 mmol/Ld of CO_2_ were removed by the biocathode of the filter press MEC [39]. The higher CO_2_ removal rate reached in the filter press MEC was justified by the current density of 91 A/m^3^, which resulted in a higher value with respect to the 19 A/m^3^ reached in the tubular MEC. The volumetric current density referred to the empty cathodic volume due to the fact that in each reactor, the same graphite granules were adopted as electrodic material. Moreover, the higher current density promoted a higher energetic consumption of the filter press MEC, which resulted in 2.36 kW·h/Nm^3^ CO_2_ being removed while the tubular reactor allowed for a consumption of 0.8 kW·h/Nm^3^. As reported in Table 5, the specific CO_2_ removal parameter, normalized for the cathodic volumetric current density, showed a higher performance was obtained by the tubular MEC, which allowed a 2.5 times higher CO_2_ removal with respect to the filter press MEC.

Regarding the nitrogen recovery performances, the comparison of the filter press MEC and the tubular MEC was performed considering the applied nitrogen load rate (NLR) applied to the anodic chamber in two different previous experiments [40,43]. As reported in Table 6, the filter press MEC showed a higher ammonium recovery rate at the lower NLR applied to the anode. On the contrary, by applying higher NLR, the ammonium recovery rate was similar in the two MEC, with a slightly higher ammonium recovery rate obtained by the tubular MEC. Moreover, even if in the filter press a higher current density was reached, the ammonium recovery rate was influenced mainly by the applied NLR to the anodic chamber, i.e., the NLR directly influences the availability of ammonium ions for the electroneutrality maintenance.

The performances of the tubular reactor are comparable with the performance obtained through the smaller scale filter press MEC; however, the obtained current densities obtained in the tubular MEC are considerably lower, indicating the good potential of the reactor at higher current densities.

## 4. Conclusions

The experimental study demonstrated the feasibility of the bioelectrochemical process for nitrogen recovery and simultaneous COD and CO_2_ removal with the utilization of a 12-L tubular geometry MEC. The MEC was operated under three different nitrogen load rates, maintaining the same organic load rate, to study the influence of the process with respect to the ammonium nitrogen content. The nitrogen load rate increase resulted in a progressive increase of the anodic COD removal, which increased from 1.8 ± 0.3 g COD/day during the first operating period to 6.3 ± 0.6 g COD/day during the third operating period; the COD removal increase was probably due to the acclimation of non-electroactive microorganisms along the process operation. The increase of the COD removal did not promote a consequent electric current increase, i.e., a slight decrease in terms of the current output was observed by increasing the nitrogen load rate. As a consequence, the coulombic efficiency of the anodic reaction decreased from 77% to 18%. The cathodic bioelectrochemical reduction of CO_2_ into CH_4_ increased during the explored operating conditions, giving almost a recovery of the current into methane (i.e., the cathode capture efficiency) during the second and third operating periods, with average values of 98% and 81%. The nitrogen load rate increase promoted ammonium migration and recovery, with a nonlinear magnitude, i.e., by increasing 5 and 30 times the nitrogen load rate with respect to the first operating period. The ammonium recovery and the corresponding ammonium contributed to the electroneutrality maintenance being increased by 10 and 100 times. An interesting effect of the nitrogen load rate increase resulted in the increase of the electro osmotic diffusion of the liquid phase from the anode to the cathode chamber. The CO_2_ removal from the cathodic chamber was slightly increased by the nitrogen load rate. By the analysis of the CO_2_ removal mechanisms, the role of the alkalinity generation resulting from the electroneutrality maintenance was underlined by the fact that almost 50% of the removed CO_2_ was promoted by the migration of ammonium or other cations (such as calcium and magnesium). The analysis of the energetic consumption of the bioelectrochemical process showed a lower energy consumption for COD and CO_2_ removal with respect the benchmark technologies: 1.2 kW·h/kg COD for activated sludge and 0.8 kW·h/Nm_3_ CO_2_ for the water scrubbing biogas upgrading technology. The ammonium nitrogen recovery energetic cost was interesting, particularly during the third operational period in which an energy consumption of 2.3 kW·h/kg N was used for the nitrogen recovery. It is also important to underline the fact that the energy consumption in the bioelectrochemical process was simultaneously utilized for COD removal, CO_2_ removal, and nitrogen removal. Moreover, additional energy recovery is offered by CH_4_ production, which is described by the energy efficiency of the process, which was 52% during the third operating period. Finally, by comparing the performances of the tubular MEC with a previous bench-scale MEC, similar performances were obtained in terms of the CO_2_ removal rate and ammonium recovery rate; however, due to the considerably lower current densities obtained in the tubular MEC, the good potential of the upscaled tubular MEC can be assessed, indicating the necessity of a current density increase.

## Figures and Tables

**Table 1:** Main bioelectrochemical parameters obtained during the three different operating periods.

Nitrogen Loading Rate (mgN/Ld)	73	365	2229
Current (mA)	190 ± 14	166 ± 10	157 ± 7
COD removed (g COD/d)	1.8 ± 0.3	4.0 ± 0.3	6.3 ± 0.6
COD removal efficiency (%)	29 ± 11	65 ± 17	89 ± 20
Coulombic Efficiency (CE, %)	77 ± 17	30 ± 4	18 ± 2
Methane production (mmol/d)	9 ± 1	18 ± 1	14 ± 2
Cathodic Capture Efficiency (CCE, %)	42 ± 8	98 ± 11	81 ± 14

**Table 2:** N removal and mass balance during the three operating conditions.

Nitrogen Loading Rate (mg N/Ld)	73	365	2229
N influent (mg N/L)	37 ± 2	241 ± 14	1341 ± 28
N effluent (mg N/L)	25 ± 2	148 ± 9	1013 ± 66
N cathode (mg N/L)	101 ± 9	674 ± 48	2094 ± 78
N removal (%)	33 ± 13	45 ± 12	36 ± 7
∆N (mg N/day)	89 ± 31	713 ± 150	3246 ± 558
N_spilled_ (mg N/day)	31 ± 3	281 ± 20	2445 ± 91
N VSS_out_ (mg/day)	84 ± 3	106 ± 4	90 ± 8
Mass balance recovery (%)	109 ± 8	80 ± 7	92 ± 9
N transported charge (mA)	2 ± 1	22 ± 2	195 ± 7

**Table 3:** Bicarbonate concentration in the different MEC liquid phases and the inorganic mass balance of the three different operating periods.

Nitrogen Loading Rate (mg·N/Ld)	73	365	2229
HCO_3_^−^ influent (gHCO_3_^−^/L)	1.17 ± 0.07	0.10 ± 0.02	0.11 ± 0.03
HCO_3_^−^ effluent (gHCO_3_^−^/L)	1.09 ± 0.07	0.49 ± 0.06	0.61 ± 0.06
HCO_3_^−^ cathode (gHCO_3_^−^/L)	10.94 ± 1.20	10.72 ± 0.56	11.39 ± 2.10
CO_2_ removal (mmol/day)	443 ± 40	453 ± 19	481 ± 38
rCH_4_ (mmol/day)	9 ± 1	18 ± 1	14 ± 2
HCO_3_^−^_spilled_ (mmol/day)	55 ± 6	73 ± 4	218 ± 40

**Table 4:** Energy consumptions for the different MEC operations and energy efficiencies of the MEC during the three operating periods.

Nitrogen Loading Rate (mg N/Ld)	73	365	2229
kW·h/kg COD	6.8 ± 0.5	2.00 ± 0.1	0.88 ± 0.08
kW·h/Nm^3^ CO2	1.12 ± 0.31	0.72 ± 0.07	0.47 ± 0.02
kW·h/kg N	388 ± 13	28 ± 4	2.3 ± 0.5
ηE (%)	17 ± 1	52 ± 2	59 ± 1

**Table 5:** Performance comparison of the tubular MEC and previous literature data for the cathodic CO_2_ removal.

	Present Study	Reference Study [39]
**CO_2_ Removal (mmol/Ld)**	51	100
**kW·h/Nm^3^ CO_2_**	0.8	2.36
**Volumetric Current Density (A/m^3^)**	19	91
**Specific CO_2_ Removal Rate (mol/Ad)**	2.7	1.1

**Table 6:** Performance comparison of the tubular MEC with previous literature data for the ammonium recovery.

Nitrogen Loading Rate (mg N/Ld)	Present Study 73	Present Study 2229	Reference Study [40] 73	Reference Study [43] 1670
**Ammonium Recovery** **(mg N/Ld)**	4	276	38	228
**Volumetric Current Density** **(A/m^3^)**	21	19	128	72
